# Supplementary material for: A high-throughput multispectral imaging system for museum specimens
Source: Commun Biol. 2022 Dec 1;5:1318. doi: 10.1038/s42003-022-04282-z (PMC9715708; doi:10.1038/s42003-022-04282-z)
Supplement: Supplementary file 2 — Supplementary Information [file 42003_2022_4282_MOESM2_ESM.pdf]

Supplementary Information for

A high-throughput multispectral imaging system for museum specimens

Wei-Ping Chan, Richard Rabideau Childers, Sorcha Ashe, Cheng-Chia Tsai, Caroline Elson, Kirsten J. Keleher, Rachel L. Hawkins Sipe, Crystal A. Maier, Andrei Sourakov, Lawrence F. Gall, Gary D. Bernard, Edward R. Soucy, Nanfang Yu, and Naomi E. Pierce

Data structure

Initial descriptive data

The layers are 740, 940, UV, UVF, F, white, whitePo1, whitePo2, F(RGB), Polarized Diff (abs), mask layer, #pixels/1cm

Processed data

These data include but are not limited to all parameters generated during image processing, gridded multispectral reflectance, wing shapes, and the measurements of body size and antennae. Detailed data structure can be found on the GitHub repository.

Group summary matrix

For a target group, the data include but are not limited to the mean and standard error of the multispectral reflectance for fore- and hindwings (dorsally and ventrally), the average wing shapes (fore- and hindwing), the basic statistics describing body and antennal morphologies. The information for the specimens included in this analysis is also recorded for the convenience of tracking back to the original specimens. Detailed data structure can be found in the GitHub repository.

Cost

The imaging pipeline we present is associated with two main costs: one for hardware, and the other for computation. For hardware, the costliest components are the modified digital SLR camera, which in our setup is a Nikon D800 purchased for $2300, and the broadband spectrally neutral white and black reference standards ($1100 for both). The remaining components, mostly LED light sources, the motorized filter wheel assembly, polarizing and UV pass filters and supporting electronic components and aluminum lightbox construction materials, come to an additional $1000, and have a much broader range of substitutes with comparable functionality to allow for cost-effective sourcing. By following the materials listed in our protocol, a comparable imaging platform could be built for a total hardware cost of less than $5000.

For the computational costs of image processing, we highly recommend that users in academic or governmental settings take advantage of increasingly accessible high-performance-computing resources which provide the most effective means of handling large amounts of raw image data.

For users who lack institutional access to such resources, commercial cloud-based computing resources from vendors such as Google or Amazon should work as well, but pricing will vary depending on the quantity and size of the species being analyzed.

Here, we provide some example estimates made using Google Cloud Computing (Google Inc.) pricing as a reasonable starting point for interested users. For a single set of 10 lycaenid-sized specimens imaged together, dorsally and ventrally, running on a single 32GB core with the accompanying 16Gb storage space needed and a paid operating system in a single month, the per specimen cost would be $0.65. However, the cost per specimen decreases with scale, as the equivalent computation costs needed to process 100 lycaenid specimens (e.g. one 6-hour job per day, 5 days per week with accompanying storage space that is cleared after every job) would be $41.57 or $0.416 per specimen. Equivalent calculations for 1000 specimens computed with 10 cores at a time and 10x more accompanying storage are $386.91 total or $0.387 per specimen.

Map of archived materials, protocols, and tutorials

To prevent potential conflicts, scripts for different purposes on the cluster and on the local machine are provided in different protocols on Protocols.io and repositories on GitHub. Here, the summary of online protocols and source codes are organized as follows. Inclusion in [Protocol] indicates the corresponding step-by -step instruction on Protocols.io; inclusion in [Cluster] indicates the script will run better on the cluster; inclusion in [Local] indicates the script is designed for local machines with relatively low CPU and memory demands.

- Raw data

https://datadryad.org/stash/share/Oz783oKYRj7apEmZg32cQq5ue5hntud3w5bID2DczI0

  - [[Folder/File name]]: descriptions
  - [[Methodology_imaging_records.csv]]: A file recording image names and the barcode of imaged specimens
  - [[Drawer_img_nef]]: Drawer images in RAW (*.NEF) format (total 35 images)
    - Five set of images: Method_1-1_dorsal, Method_1-1_ventral, Method_1-2_dorsal, Method_1-2_ventral, Method_1-r_ventral (with a scale bar placed upside-down)
  - [[Drawer_img_tiff]]: Drawer images in linearized 16-bit (*.tiff) format (total 35 images)

- [[manual_bounding_box_par]]: Manually corrected bounding boxes
- [[spp_img_inspection]]: Specimen images for visualization (*.jpg)
    - [[Problematic]]: Those problematic ones that need to be manually corrected
- [[spp_img_reMask_tiff_done]]: Specimen images (*.tiff) after the mask correction
- [[spp_first_level_product]]: The initial descriptive data or 'first-level products' (*AllBandsMask.mat). Find **Methods** for the detailed data structure
- [[spp_RGB_Imgs]]: Images used for manual fore-and hindwing segmentation
    - [[Seg_done]]: Done images (*.jpg)
    - [[Segmented]]: The fore-and hindwing segmentation parameters (*.json)
- [[spp_segmentation_analysis]]: Segmented images after inspection and manual correction
    - [[wing_segmentation_img]]: The visualizations of image segmentation (*.jpg)
    - [[wing_shape_morph-seg]]: The results of image segmentation (*morph-seg.mat)
    - [[morphology_analysis_spp_preference_table_template.csv]]: A table generated according to the images in the 'wing_segmentation_img' folder, which is later used for inspection
    - [[morphology_analysis_spp_preference_table.csv]]: The result after manual inspection, which records the condition of different body parts of a specimen
    - [[reflectance_table]]: The reflectance data for all body parts of all specimens
- [[spp_wing_grids_generation]]: Generate wing grids and processed data
    - [[inspect_imgs]]: The visualization (*.jpg) of wing grids (no correction was needed in these results)
    - [[spp_wing_parameters]]: Processed wing data. The original folder name is kept here.
    - [[wing_matrix_visualization]]: The summarized multispectral reflectance (NIR [740], fNIR [940], F, FinRGB, PolDiff, UV, UVF, white, whitePol1, whitePol2) according to wing grids.
- [[spp_second-level_product]]: The processed "second-level products". (*_d-v_gridsPars*.mat). Find **Methods** for the detailed data structure
- [[group_summary]]: The summary statistics for specified groups
    - [[specimen_groups.csv]]: A table specifying groups
    - [[specimen_groups_group_barcode_list.json]]: The group table in json format

  - [[summary_matrices]]: The summary results according to the group table (*._summary.mat)
  - [[summary_visualization]]: The summary visualization for each group (*.png)
  - [[shp_tail_adv_vis]]: Replot wing shape and tails by scripts for advanced visualization (*.png)
  - [[tail_summary_visualization]]: Replot tails by scripts for advanced visualization (*.png)

- Blueprints and materials (Fig. 6)

[Protocol] https://www.protocols.io/private/2E2FB268F7AF11EBB05F0A58A9FEAC02

- Bash scripts and shell scripts running on the cluster

[Cluster] https://github.com/weipingchan/Bash_scripts_methodology_paper

- Image preprocessing to derive initial descriptive data for museum archiving

[Protocol] https://www.protocols.io/private/DEF29A74E44E11EB96DA0A58A9FEAC02

[Cluster] https://github.com/weipingchan/single_img_processing

  - Inspection and manual correction of specimen bounding box (Fig. 3d)

  [Local] https://github.com/weipingchan/Drawer_img_manual_define_bounding_boxes

  - Inspection and manual correction of mask for background removal (Fig. 9a)

  [Local] commercial painting software, such as Adobe Photoshop

- Data preparation and processing for color and shape quantification

[Protocol] https://www.protocols.io/private/DEF29A74E44E11EB96DA0A58A9FEAC02

  - Body-part segmentation (Fig. 9c panels at right)

    manually defined fore-hindwing segmentation data

    [Local] https://github.com/weipingchan/body-seg_distribute

    Segmentation

    [Cluster] https://github.com/weipingchan/basic_segmentation

    Inspection and manual correction of primary landmarks (Fig. 9b)

    [Local] https://github.com/weipingchan/manual_landmark_correction

- Multispectral reflectance at wing-size level (as table format; Fig. 9d)

[Protocol] https://www.protocols.io/private/F3292DF1FE0211EB878B0A58A9FEAC02

[Cluster] https://github.com/weipingchan/multispectral_reflectance_wing-size_level

- Dorsal-ventral side analyses (Fig. 4)

[Protocol] https://www.protocols.io/private/F3292DF1FE0211EB878B0A58A9FEAC02

[Cluster] https://github.com/weipingchan/dorsal_ventral_analysis

- Inspection and manual correction of secondary landmarks (Fig. 1d)

[Local] https://github.com/weipingchan/manual_wing_grid_correction

- Visualization (Fig. 1g-h & Fig.5)

[Protocol] https://www.protocols.io/private/F3292DF1FE0211EB878B0A58A9FEAC02

- Multispectral reflectance at wing-pattern level with wing shape summary

[Local] https://github.com/weipingchan/dorsal_ventral_summary

- Advanced visualization for wing shapes and tails (**Methods**)

[Local] https://github.com/weipingchan/replot_tail_and_avg_shapes
