# Supplementary material for: A high-throughput multispectral imaging system for museum specimens
Source: Commun Biol. 2022 Dec 1;5:1318. doi: 10.1038/s42003-022-04282-z (PMC9715708; doi:10.1038/s42003-022-04282-z)
Supplement: Supplementary file 3 — Reporting Summary [file 42003_2022_4282_MOESM3_ESM.pdf]

nature portfolio

Corresponding author(s): Wei-Ping Chan, Naomi E.Pierce

Last updated by author(s): 11/10/ 2022

Reporting Summary

Nature Portfolio wishes to improve the reproducibility of the work that we publish. This form provides structure for consistency and transparency in reporting. For further information on Nature Portfolio policies, see our Editorial Policies and the Editorial Policy Checklist.

Statistics

For all statistical analyses, confirm that the following items are present in the figure legend, table legend, main text, or Methods section.

n/a	Confirmed	
☐	☒	The exact sample size (*n*) for each experimental group/condition, given as a discrete number and unit of measurement
☐	☒	A statement on whether measurements were taken from distinct samples or whether the same sample was measured repeatedly
☐	☒	The statistical test(s) used AND whether they are one- or two-sided<br>*Only common tests should be described solely by name; describe more complex techniques in the Methods section.*
☒	☐	A description of all covariates tested
☒	☐	A description of any assumptions or corrections, such as tests of normality and adjustment for multiple comparisons
☐	☒	A full description of the statistical parameters including central tendency (e.g. means) or other basic estimates (e.g. regression coefficient) AND variation (e.g. standard deviation) or associated estimates of uncertainty (e.g. confidence intervals)
☒	☐	For null hypothesis testing, the test statistic (e.g. *F*, *t*, *r*) with confidence intervals, effect sizes, degrees of freedom and *P* value noted<br>*Give P values as exact values whenever suitable.*
☒	☐	For Bayesian analysis, information on the choice of priors and Markov chain Monte Carlo settings
☒	☐	For hierarchical and complex designs, identification of the appropriate level for tests and full reporting of outcomes
☒	☐	Estimates of effect sizes (e.g. Cohen's *d*, Pearson's *r*), indicating how they were calculated

Our web collection on statistics for biologists contains articles on many of the points above.

Software and code

Policy information about availability of computer code

Data collection	Matlab 2021a, Python 3.7.0, Mathematica 12.3, DigiCamControl 2.0.0, DCraw 9.28, rclone, Adobe Photoshop 2021, Adobe Bridge 2021
Data analysis	R 4.0.2

For manuscripts utilizing custom algorithms or software that are central to the research but not yet described in published literature, software must be made available to editors and reviewers. We strongly encourage code deposition in a community repository (e.g. GitHub). See the Nature Portfolio guidelines for submitting code & software for further information.

Data

Policy information about availability of data

All manuscripts must include a data availability statement. This statement should provide the following information, where applicable:

- Accession codes, unique identifiers, or web links for publicly available datasets
- A description of any restrictions on data availability
- For clinical datasets or third party data, please ensure that the statement adheres to our policy

All raw data, pipelines, and code are archived and available in the on-line repository

Human research participants

Policy information about studies involving human research participants and Sex and Gender in Research.

Reporting on sex and gender	NA
Population characteristics	NA
Recruitment	NA
Ethics oversight	NA

Note that full information on the approval of the study protocol must also be provided in the manuscript.

Field-specific reporting

Please select the one below that is the best fit for your research. If you are not sure, read the appropriate sections before making your selection.

☐ Life sciences ☐ Behavioural & social sciences ☒ Ecological, evolutionary & environmental sciences

For a reference copy of the document with all sections, see nature.com/documents/nr-reporting-summary-flat.pdf

Ecological, evolutionary & environmental sciences study design

All studies must disclose on these points even when the disclosure is negative.

Study description	We used more than 10,000 pinned museum specimens to develop and test the imaging system and our imaging processing pipeline.
Research sample	In this paper, we presented results based on seventeen exemplar specimens representing seven families of Lepidoptera.
Sampling strategy	To emphasize the general applicability of our approach, we selected specimens to image from different lepidopteran families with highly diverse wing shapes and sizes. We also selected several species to highlight special multispectral properties we observed during the development of our system.
Data collection	The exemplar data shown in this paper were imaged and processed by W.-P. Chan and S. Ashe.
Timing and spatial scale	The raw images shown in this paper were taken on August 6, 2021. The multiple steps of image processing were done intermittently from that time until September 27, 2021.
Data exclusions	No data were excluded
Reproducibility	We provided all raw data and intermediate products at all stages. The results can be reproduced by following our processing pipeline.
Randomization	The rationale for grouping has two main goals: (1) maximize diversity by including highly diverse specimens; (2) maximize taxonomic spread across families to provide insights based on our exemplar sampling
Blinding	Our paper is aimed at showing the details and applications of our system, so blinding is not applicable here.

Did the study involve field work? ☐ Yes ☒ No

Reporting for specific materials, systems and methods

We require information from authors about some types of materials, experimental systems and methods used in many studies. Here, indicate whether each material, system or method listed is relevant to your study. If you are not sure if a list item applies to your research, read the appropriate section before selecting a response.

Materials & experimental systems

n/a	Involved in the study
☒	☐ Antibodies
☒	☐ Eukaryotic cell lines
☒	☐ Palaeontology and archaeology
☐	☒ Animals and other organisms
☒	☐ Clinical data
☒	☐ Dual use research of concern

Methods

n/a	Involved in the study
☒	☐ ChIP-seq
☒	☐ Flow cytometry
☒	☐ MRI-based neuroimaging

Animals and other research organisms

Policy information about studies involving animals; ARRIVE guidelines recommended for reporting animal research, and Sex and Gender in Research

Laboratory animals	The study did not involve lab animals.
Wild animals	The study was carried out with dead, pinned museum specimens that had been collected in the wild.
Reporting on sex	Sex was not considered in the development of our paper.
Field-collected samples	The study is based on specimens that were originally collected in the field, but are now curated within the Museum of Comparative Zoology entomology collections.
Ethics oversight	No ethical approval was required since the insects studied were dead, pinned specimens from the collection of the Museum of Comparative Zoology.

Note that full information on the approval of the study protocol must also be provided in the manuscript.
